# A New Anæsthetic

**Published:** 1891-10

**Authors:** 


### SELECTIONS.

### A New Anæsthetic.

It is a long time ago since John Hunter, when cutting into the cock's comb, noticed the anæsthetic effects of cold. Although his primary object was to restrain bleeding, he nevertheless remarks that the operation was apparently painless. Dr. Richardson also noticing the benumbing effects of cold, devised his apparatus for its artificial production by spraying ether on the part to be operated on, but from cause ether spray fell into disfavor with surgeons, and was finally superseded by cocaine as a local anæsthetic. Cocaine, however, has not fulfilled all the promises held out for it by its advocates; it is not perfectly inocuous, and it has been blamed for delaying the healing of the cut surfaces of the part anæsthetized by it. The search for a new anæsthetic, local in its action and non-poisonous, was recommenced, and, if we accept M. Monnet's statements, we have in the chloride of ethyl all that is sought. Already it has found an advocate in Dr. C. Redard, Clinical Professor at the Geneva School of Dentistry, who speaks highly in its favor.

Chloride of ethyl ($C_2H_5Cl.$), is a colorless mobile liquid, having a peculiar and pleasant odor, and a sweetish burning taste. It boils at 12.5°, at 0° possesses a specific gravity of 0.9214. It is slightly soluble in water, but dissolves readily in alcohol. It is sent out for medicinal use in hermetically sealed glass tubes containing a little more than two drachms each. When required for use the point of the tube is snipped off and the warmth of the operator's hand is sufficient to cause a very fine jet of the chloride to be projected on the part to be anæsthetized. No apparatus is required—a great advantage. Up to the present its use has been confined to dentistry and as an external application in neuralgic affections, but there is little doubt that in a short time its value will be tested in general surgery. Although the use of the chemical as an anæsthetic is new, the chloride is one of our oldest preparations. Of course, it was not known as chloride of ethyl, for the name "ethyl" was the invention of Berzelius, and the word "chloride" a little later (1812) by Sir H. Davy. But in the beginning of the fifteenth century, at the end of the ecclesiastical era in medicine, and the dawn of the Renaissance, when Brunelleschi commenced the task of reviving the love of the beautiful by copying Greek architecture and Greek statuary, even prior to the birth of Linacre, Basil Valentine, by distilling common salt and spirits of wine, having, as he says, previously united them, obtained an alcoholic solution of the chloride. "Thus is the *spiritus salis et vini* prepared." And Johann Rodolph Glauber, better known as the maker of the "sal mirabile," (Glauber's salt), in 1648 wrote: "When dephlegmated spirit of wine is poured in strong spirit of salt and digested for a long time, the spirit of wine makes a separation and kills its *sal volatile* so that a fine clear *oleum vini* swims on the top, which is not the least potent of the cordials."—*Med. Press and Circular.*

